# News and Coming Events

**Published:** 1909-05-22

**Authors:** 


### News and Coming Events.

Dr. Stuart McDonald, F.R.C.P.E., the well-known Edinburgh pathologist, has recently been appointed Professor of Pathology in the College of Medicine, Newcastle-on-Tyne, which is the Medical School of the University of Durham, and Pathologist to the Royal Victoria Infirmary, Newcastle.

According to the New York correspondent of the *Times*, telegraphing on May 16, the fight against tuberculosis in that city continues with unabated vigour. Chicago recently, by a referendum, sanctioned the levy of a tax for the erection and maintenance of a sanatorium for consumptives, and Governor Hughes has just signed Bills authorising New York City and the counties of the State to establish similar hospitals. The same informant states that the Health Commissioner for New York publishes figures showing that the death-rate for the city from tuberculosis in 1908 was the lowest on record. The number of cases reported during the last two years has increased, but this the Commissioner attributes to earlier and more complete registration.

The fifty-seventh annual meeting of the governors of the Hospital for Sick Children, Great Ormond Street, was held at the hospital on May 13, the Duke of Fife, president, in the chair. The report of the committee naturally dwelt upon the completion and opening of the new out-patient department. In-patients in 1908 numbered 3,449, or 274 more than in 1907; and the daily average number of resident patients rose from 182 to 189. Out-patients, on the other hand, showed a falling-off. The income was £24,188, and the expenditure £23,474. The midsummer fair and fête at Olympia will be opened on June 23.

A festival banquet in aid of the Maintenance Fund of the Mount Vernon Hospital for Consumption and Diseases of the Chest was held at the Hôtel Cecil last week. Lord Clifden, Deputy-Chairman, presided, and in proposing "Prosperity to the Mount Vernon Hospital," he stated that the number of beds now occupied was 226, including 120 at Hampstead, and 106 at Northwood. During the last year 3,000 new patients were treated in Fitzroy Square. The treatment at Hampstead compared favourably in its results with that at any similar institution. The actual expenditure required was £18,000 per annum, and last year the average expenditure per patient per week was £1 5s. 4d. Mr. Stedall, in acknowledging the toast, referred to the recent establishment of a special ward of eight beds for treatment by serum therapy. The Secretary announced subscriptions amounting to £2,180 16s.

The seventy-seventh annual meeting of the British Medical Association will be held at Belfast from July 23 to July 31, 1909. The President-elect is Sir William Whitla, M.D., LL.D., Professor of Materia Medica and Therapeutics, Queen's College, Belfast. The presidential address will be delivered on Tuesday, July 27, and the sections will meet on the three following days. The annual representative meeting will begin on Friday, July 23, 1909. The address in medicine will be delivered by Dr. Robert W. Philip, M.D., F.R.C.P.Edin., Physician to the Royal Infirmary, Edinburgh. The address in surgery will be delivered by Mr. A. E. J. Barker, F.R.C.S., Professor of Surgery, University College, London. The address in obstetrics will be delivered by Sir John W. Byers, M.D., Professor of Midwifery and Diseases of Women, Queen's College, Belfast. The popular lecture will be delivered by Dr. J. A. Macdonald, Physician to the Taunton and Somerset Hospital, Chairman of the Representative Meetings.

The Danish Royal Scientific Society has admitted as a foreign member Dr. Dreyer, Professor of Pathology in the University of Oxford.

At a meeting of the Welsh University Court, held at Mold on May 14, Sir Isambard Owen was again re-elected Senior Deputy Chancellor and Sir John Williams was elected Junior Deputy Chancellor.

The new out-patients' department at the Royal Portsmouth Hospital was opened on May 11. This department has been erected at a cost of £4,000, which was given by Mr. Woolmer White, of Havant.

The honorary degree of LL.D. of the McGill University of Montreal will be conferred on Dr. George A. Gibson, M.D., D.Sc., F.R.C.P.(Edin.), the distinguished Edinburgh physician at the Medical Convocations on June 9. Dr. Gibson gave the Inaugural Address of the Medical Faculty of the McGill University last September.

The course of four lectures on Statistical Methods in Physiology and Medicine at the London Hospital Medical College, by Mr. Major Greenwood, jun., M.R.C.S., L.R.C.P., is just commencing. The first lecture was on Friday, May 21, at 4.30 p.m., and the course will be continued on June 4, 11, and 18. The lectures are open to students of the University of London, and others on presentation of their cards.

The Harben Lectures of the Royal Institute of Public Health will be delivered this year by Professor R. Pfeiffer, Director of the Hygiene Institute, Breslau, in the Lecture Room of the Institute, at 6 o'clock, at 37 Russell Square, London, W.C. The first lecture, on the Importance of Bacteriolysins in Immunity, will be given on Monday, June 21; the second, on Endotoxins and Anti-endotoxins, on Wednesday, June 23; and the third, on the Problem of Virulence, on Friday, June 25.

